# A Transient Dopamine Signal Represents Avoidance Value and Causally Influences the Demand to Avoid

**DOI:** 10.1523/ENEURO.0058-18.2018

**Published:** 2018-05-15

**Authors:** Katherine J. Pultorak, Scott A. Schelp, Dominic P. Isaacs, Gregory Krzystyniak, Erik B. Oleson

**Affiliations:** University of Colorado Denver, Denver, CO 80217

**Keywords:** avoidance, behavioral economics, dopamine, motivation, negative reinforcement, voltammetry

## Abstract

While an extensive literature supports the notion that mesocorticolimbic dopamine plays a role in negative reinforcement, recent evidence suggests that dopamine exclusively encodes the value of positive reinforcement. In the present study, we employed a behavioral economics approach to investigate whether dopamine plays a role in the valuation of negative reinforcement. Using rats as subjects, we first applied fast-scan cyclic voltammetry (FSCV) to determine that dopamine concentration decreases with the number of lever presses required to avoid electrical footshock (i.e., the economic price of avoidance). Analysis of the rate of decay of avoidance demand curves, which depict an inverse relationship between avoidance and increasing price, allows for inference of the worth an animal places on avoidance outcomes. Rapidly decaying demand curves indicate increased price sensitivity, or low worth placed on avoidance outcomes, while slow rates of decay indicate reduced price sensitivity, or greater worth placed on avoidance outcomes. We therefore used optogenetics to assess how inducing dopamine release causally modifies the demand to avoid electrical footshock in an economic setting. Increasing release at an avoidance predictive cue made animals more sensitive to price, consistent with a negative reward prediction error (i.e., the animal perceives they received a worse outcome than expected). Increasing release at avoidance made animals less sensitive to price, consistent with a positive reward prediction error (i.e., the animal perceives they received a better outcome than expected). These data demonstrate that transient dopamine release events represent the value of avoidance outcomes and can predictably modify the demand to avoid.

## Significance Statement

Dopamine is thought to play a crucial role in reward learning and directing actions toward beneficial outcomes. While the avoidance of harmful stimuli is similarly pertinent to an organism’s survival, the role of dopamine in avoidance remains controversial. Using *in vivo* electrochemistry, we observed that dopamine concentration decreased when the effort (lever presses) required to avoid electrical footshock increased. We also found that increasing dopamine at an avoidance predictive cue decreased avoidance, consistent with a negative prediction error. In contrast, increasing release at successful avoidance increased avoidance, consistent with a positive prediction error. These data demonstrate that transient dopamine release events represent the value of avoidance outcomes and capably modify avoidance.

## Introduction

Dopamine release events within the mesocorticolimbic pathway actively guide behavior in positive reinforcement, where behavior is strengthened by the occurrence of a rewarding event (Schultz et al., 1997; Glimcher, 2011). A growing literature shows that a transient dopamine signal represents reward value and causally modifies the valuation of reward (Schelp et al., 2017; Schultz et al., 2017). It was first demonstrated that bursts of dopamine neural activity occur when animals are presented with an unexpected reward or a reward predictive cue but are suppressed when reward is withheld. These observations led to the view that dopamine neurons represent a reward prediction signaled by the cue (Schultz et al., 1997). It was then shown that the extent of the dopamine response corresponds to predicted reward magnitude (Tobler et al., 2005; Enomoto et al., 2011; Lak et al., 2014; Stauffer et al., 2014; Schelp et al., 2017), suggesting that dopamine actually represents a value prediction signaled by the cue (Schelp et al., 2017; Schultz et al., 2017).

An extensive body of literature suggests that dopamine also plays a role in avoiding harm (i.e., negative reinforcement). Dopamine terminal lesions (McCullough et al., 1993) and dopamine receptor antagonists (Arnt, 1982; Wadenberg et al., 1990) impair avoidance of electrical footshock while genetic restoration of dopamine within the striatum of otherwise dopamine-deficient mice is necessary to maintain avoidance (Darvas et al., 2011). Microdialysis studies have demonstrated that striatal dopamine concentration is increased during the avoidance of footshock and in postsurgical pain relief (McCullough et al., 1993; Navratilova and Porreca, 2014). More recently, studies using fast-scan cyclic voltammetry (FSCV) showed that transient increases in dopamine concentration occur in response to cues signaling the opportunity to avoid electrical footshock (Oleson et al., 2012; Gentry et al., 2016). While these studies indicate a role for dopamine in avoidance, it remains controversial whether dopamine plays a role in the valuation of avoidance. Indeed, a notable electrophysiology study concluded that dopamine neurons predominately encode the value of positive reinforcement (Fiorillo, 2013). Similarly, a recent FSCV study found that transient dopamine release events encode ultrasonic vocalizations associated with a positive, but not a negative affective state, indicating that the behavior response to negatively reinforcing stimuli is regulated by distinct brain areas (Willuhn et al., 2014).

To investigate the role of dopamine in the valuation of avoidance, we used a behavioral economics task in which rats were presented with an avoidance predictive cue and provided the opportunity to avoid the onset of electrical footshock by responding on a lever. We then increased the price of avoidance by increasing the number of lever presses required to avoid footshock at fixed intervals over the course of a session. To measure changes in price sensitivity, we generated demand curves to model avoidance as a function of price. The rate at which these demand curves decay depicts price sensitivity and allows for inference of the worth an animal places on an outcome.

If, as in reward seeking (Schultz et al., 2015; 2017), dopamine represents the value to avoid aversive outcomes, than dopamine concentration at avoidance-predictive cues should decrease as the price to avoid increases. Furthermore, optically increasing release at cue presentation should increase price sensitivity. In theory, artificially augmenting release at cue presentation would lead the animal to expect a better outcome. When the animal’s expectation is negatively violated by the recurrence of the same amplitude of footshock, the animal becomes more sensitive to price because the worth of avoidance is diminished. By contrast, optically increasing release at successful avoidance should increase the demand to avoid. In this case, we infer that the worth of avoidance is increased because the animal perceives that they received a better bargain than predicted.

As hypothesized, dopamine scaled inversely with cost; however, both dopamine and avoidance were concurrently attenuated at session onset. Augmenting dopamine release at an avoidance predictive cue rendered animals more sensitive to avoidance costs. We attribute this finding to a negative reward prediction error, whereby the animal perceives they received a worse value than anticipated when they receive the same amplitude of electrical footshock. Increasing release at successful avoidance made animal less sensitive to avoidance costs. We attribute this finding to a positive reward prediction error, whereby the animal perceives they received a better value than anticipated despite still receiving footshock in other trials. From these data, we conclude that dopamine release events represent the value of avoidance and casually modify the demand to avoid.

## Materials and Methods

### Subjects and surgeries

Male Long–Evans rats provided by Charles River Laboratories as well as Transgenic rats (LE-Tg(TH-Cre)3.1Deis) expressing Cre-recombinase under the tyrosine hydroxylase (TH) promotor (TH::Cre±) supplied by Rat Resource and Research Center were used as subjects. Rats were singly housed and maintained on a 12/12 h light/dark cycle with the dark cycle beginning at 10 A.M. All experiments were conducted during the dark (active) cycle with food, water, and crinkle paper enrichment provided *ad libitum*. Surgeries were conducted at 300–350 g using Kopf stereotaxic equipment with rats anesthetized at 5% isoflurane and maintained at 2 ± 1%. For FSCV, rats were implanted with a microdialysis guide cannula (BASi) targeted at the nucleus accumbens (NAcc) core (+1.3 AP, +1.4 ML) of the right hemisphere and a contralateral Ag/AgCl reference electrode. For optogenetic surgeries, rats were unilaterally transfected with 4 µl of a Cre-dependent virus (rAAV2/EF1a-DIO-hChR2(H134R)-EYFP; UNC Vector Core) targeted at the ventral tegmental area (VTA). A total of 1-µl viral aliquots were infused at four areas surrounding the VTA (−5.2 AP and −6.0 AP, −0.5 ML, −7.4 AP and −8.4 AP) at a rate of 50 nl/min. A fiber optic cannula (ThorLabs; 200 µm core) was then implanted unilaterally, directed at the VTA (−5.6 AP, +0.5 ML, −7.9 DV). These methods were used for both the transgenic rats as well as wild-type (WT) counterparts, which served as controls in optogenetic experiments. After surgery, rats were given >3 d to recover during which time each received a daily 3-ml intraperitoneal injection of a 1% carprofen solution to reduce inflammation and postoperative pain. All animal procedures were performed in accordance with the University of Colorado Denver animal care committee's regulations.

### Behavioral tasks

Rats were maintained on a daily training schedule (7 d/week) within operant boxes (Med-Associates) outfitted with footshock grid floors. Rats were first trained to escape electrical footshock with each daily escape-only session lasting 15 min. At the onset of each session, both an active and inactive lever were extended, a cue light placed above the active lever was illuminated and 0.5-mA electrical current (i.e., footshock) was applied to the grid floor of the operant chamber. A response on the active lever terminated the ongoing foot shock and allowed the rat to escape into a 30-s safety period accompanied by a tone, while a response on the inactive lever had no effect. This safety signal played for the entirety of the safety period, the end of which coincided with the onset of the next escape trial. In this initial training task, escape behavior was shaped by a researcher who could simulate a lever response using a wireless keyboard, thereby reinforcing a series of behaviors that would lead to the acquisition of operant escape. The experimenter first shaped the animal to the quadrant of the operant box containing the extended lever. They then reinforced rearing behavior in front of the lever before finally requiring the rat to respond on the lever to self-terminate electrical footshock. This training persisted until the animal demonstrated an association between lever response and shock termination through consistent and researcher-independent escape (>20 sequential escape responses).

Following the acquisition of escape behavior animals were moved into a daily 1-hr avoidance task in which reinforcement was maintained under a fixed ratio1 (FR1) schedule of reinforcement. This task added the potential for an avoidance outcome. Each session was initiated by the extension of both an active an inactive lever and the illumination of a cue light placed directly above the active lever. In each trial, rats were given 1s from cue presentation to respond on the active lever to successfully avoid footshock. If rats failed to respond, recurrent footshock (0.5 s, 0.5 mA every 1 s) was applied until a lever response was made to escape further shock. Responses made on the inactive lever had no effect on behavioral outcome. Rats remained on this task until they consistently performed (more than or equal to three sessions) ≥50% avoidance.

**Figure 1.**
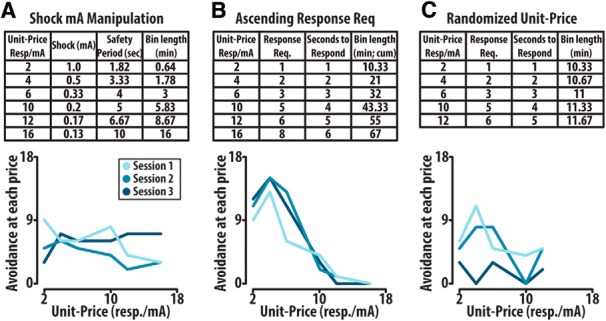
Unit-price randomization and manipulation of unit-price through changing mA shock avoided prevents establishment of baseline avoidance performance during a within session design in which unit-price epoch duration was modulated to allow 20 avoidance opportunities at each unit-price. ***A***, The first six unit-prices, achieved through decreases in the mA of shock avoided (upper), were presented in descending order to animals and avoidance behavior was assessed. Representative avoidance with mA shock manipulation demonstrates variability and failure of avoidance to scale with increasing price (lower). ***B***, Ascending unit-price through increase in response requirement (upper) demonstrates a unique, but stable avoidance demand curve with increasing unit-price (lower). ***C***, The first five unit-prices, achieved through increasing response requirement (upper), were randomly presented to animals, and avoidance behavior was assessed; representative avoidance behavior demonstrates variability between sessions and failure to establish a baseline of avoidance (lower).

**Figure 2.**
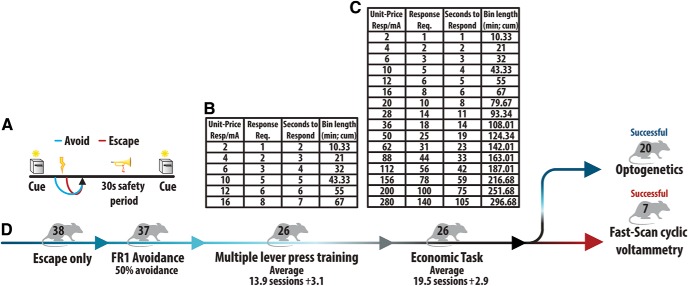
Shock avoidance paradigm and behavioral economics methodology. ***A***, An avoidance predictive cue, characterized by the presentation of a lever and the illuminator of a cue light, is presented to signal the opportunity for an animal to respond on the lever to avoid. If a response is made within a set interval (***B***, ***C***, column 3), the animal does not receive footshock. This is considered an avoidance (blue). If the animal fails to meet the response requirement with a set interval, the animal receives recurrent footshock until the full response requirement is met and the animal escapes further shock (red). ***B***, To increase the cost of avoidance, unit-price (column 1: defined as response requirement/mA shock) increases through an increase in response requirement (column 2). Animals were initially presented six unit-prices to train multiple lever press responding with additional time provided to meet higher response requirements before shock onset (column 3). The epoch length increases in duration to allow for 20 avoidance opportunities at every unit-price (column 4). ***C***, On acquisition of multiple lever press responding, animals were moved to the economic based task wherein 16 ascending unit-prices were presented. Here, seconds to respond before shock onset (column 3) was restricted relative to the six unit-price training task. ***D***, Timeline depicting animals’ progression through active operant avoidance training. A total of 38 animals were first trained to respond to escape unavoidable footshock. The 37 animals that fully acquired escape behavior moved into a FR1 avoidance task until rats consistently (more than or equal to three sessions) performed avoidance at ≥50% avoidance performance. On acquisition of avoidance behavior, animals were trained to meet increasing response requirements across six unit-price epochs. Of the 37 animals introduced to this task, 26 demonstrated avoidance at more than or equal to three unit-prices, taking an average of 13.9 ± 3.1 sessions to acquire, and proceeded to the next task. Animals were moved into an economic footshock avoidance task with 16 ascending unit-prices. Here, seconds (column 3) to respond before shock onset was restricted relative to the training task. On establishing a stable, baseline rate of avoidance (average, 19.5 ± 2.9 sessions), animals were placed either in the optogenetics or FSCV group.

**Figure 3.**
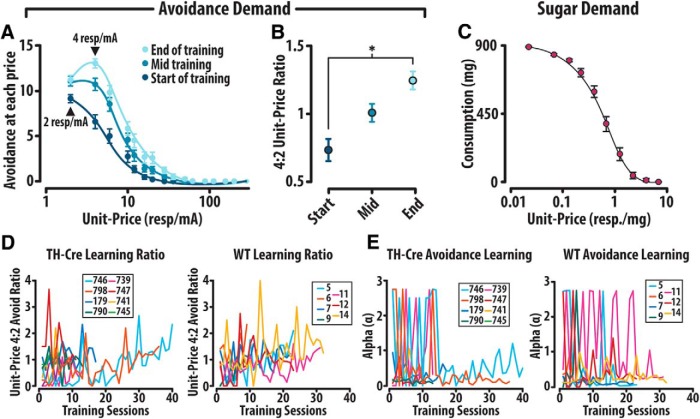
Attenuation of avoidance at 2 resp/mA develops during acquisition of the economic shock avoidance task. ***A***, As animals acquired this economic shock avoidance task (compare Fig. 1*C*), an attenuation of avoidance at the lowest unit-price (2 resp/mA) developed relative to responding at the second unit-price (4 resp/mA). ***B***, The ratio between avoidance at 4 resp/mA and avoidance at 2 resp/mA significantly increased as animals established baseline rates of performance. ***C***, Demand curves depicting economic food seeking does not demonstrate attenuation of consumption at session onset. ***D***, The ratio of avoidance at 4:2 resp/mA increases and stabilizes over the course of training in both the TH-Cre and WT group. ***E***, α, a measure of avoidance demand, similarly stabilizes over the course of training. Error bars are mean ± SEM.

After successful acquisition of 50% avoidance under an FR1 schedule, rats moved into a behavioral economics-based shock avoidance task. Here, the unit-price (response requirement/mA shock avoided) increased throughout each session by increasing the response requirement to both avoid or escape footshock. Within this task, the unit-price epoch duration increased in length to allow 20 avoidance opportunities and provide sufficient time to meet increasing response requirements as well as to account for safety period duration following avoidance or escape responses. (Fig. 1*A*, column 4). Failure to meet the response requirement on the active lever within the allotted time (Fig. 1*A–C*, column 3) resulted in the onset of a 0.5 s, 0.5-mA footshock and reset any lever responses made before footshock onset to zero. Footshock recurred until the response requirement was fully met or 15 sequential footshock were received and the session terminated. Similarly, 20 sequential escape responses resulted in session termination. Rats were first trained to perform multiple lever presses to avoid footshock across six unit-prices (Fig. 2*B*). Once animals demonstrated independent multiple lever press responding, they were moved into an extended, 16 unit-price economic task until they acquired stable response output across daily sessions (Fig. 2*C*). Acquisition was defined by rats reaching a final unit-price (within a range of three price points) for three consecutive days, without showing any ascending or descending trend (Fig. 3*D*,*E*).

Unit-price was additionally manipulated through changes in the mA of shock received. Within this task, the response requirement was held constant at an FR2 and rats were given 2 s to respond before the onset of a 0.5-s footshock across six unit-prices (Fig. 1*A*, upper). Within this task animals were presented with descending unit-prices (ascending mA shock) to prevent avoidances in higher unit-prices due to elevated mA shock amplitude in lower unit-prices. Safety period duration was additionally modulated to address opportunity cost confounds in the response requirement task and unit-price epoch duration was similarly modulated to allow for 20 avoidance opportunities at each unit-price. Utilizing this task, response output failed to scale in response to changing unit-price (Fig. 1*A*, lower).

We further attempted to address order effect confounds within the ascending response requirement task by randomizing the presentation of five unit-prices within a single session (Fig. 1, upper). Randomization of unit-price resulted in inconsistent behavioral output and preemptive extinction of avoidance (lower).

Within all tasks, the cue was defined as the presentation of a lever and the illumination of a light above the lever which was also accompanied by white noise. Both an escape or avoidance response resulted in the retraction of the lever, the dimming of the light, termination of the white noise, as well as the beginning of the 30-s safety tone and illumination of a secondary house light for the duration of the safety period.

### FSCV

FSCV recordings (*n* = 7) were performed during the economics-based task. For these recordings, glass carbon-fiber electrodes were lowered into the NAcc using micromanipulators (University of Illinois at Chicago; Schmidt) and locked into place at a depth where transient dopamine release events were apparent. Electrodes were first cycled at 60 Hz *in vivo* for 30–40 min before being reduced to 10 Hz for data collection. An initial wave form (−0.4 to 1.3 V, tarheelCV filtered with cutoff frequency of 2 kHz for a scan rate of 400 V/s) was applied which allowed for the detection of dopamine via FSCVs taken every 100 ms. To extract the dopamine component, principle component regression (PCR) was applied to the raw voltammetric data as previously described (Heien et al., 2004). Specifically, DA and pH were resolved from the FSCV recordings using recording-specific training sets (*n* = 7/anylate) to produce pH background-subtracted (10 consecutive scans) dopamine concentration files. To increase the validity of calibration factors for dopamine assessment, we applied a recently developed computational model (Roberts et al., 2013) designed to calculate calibration factors for individual electrodes by applying known constants to background current values from each *in vivo* recording. By replicating Roberts et al. (2013), using 10 electrodes, we obtained a set of empirical values using multiple linear regression analysis. Our lab-specific coefficients are: α = 4.71e^−5^, β = 17.185, γ = 8.324, δ = −0.656. Using these coefficients, we can calculate calibration factors for individual electrodes used *in vivo* by simply entering the observed total background current and the switching potential used for each individual recording. For additional information see the supplemental information of Schelp et al. (2017).

**Figure 4.**
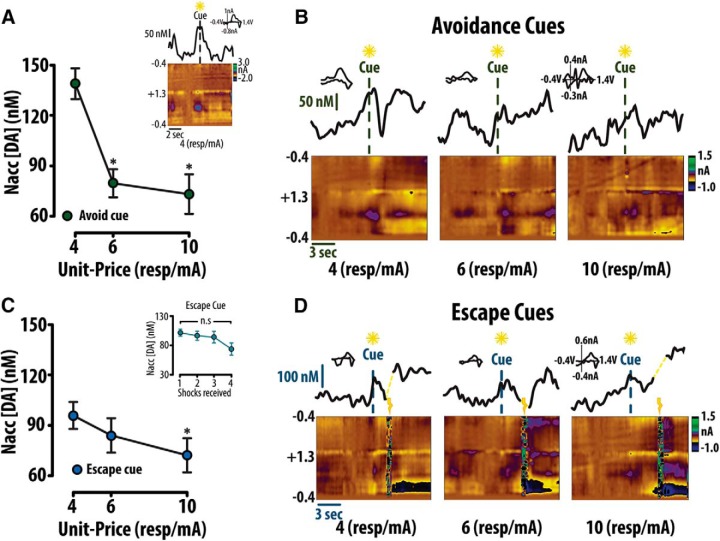
Accumbal dopamine concentration scales as a function of unit-price. ***A***, The concentration of dopamine at cues preceding avoidance at unit-prices 4, 6, and 10 resp/mA scale inversely to unit-price. Representative avoidance cue trial (inset). ***B***, Average color plots (bottom) and dopamine concentration traces (top) depicting avoidance cues demonstrate this trend. ***C***, Conversely, the concentration of dopamine at cues preceding escape decreased with increasing unit-price, while the concentration at cues preceding 1, 2, 3, and 4 shocks received across all unit-prices demonstrated no sensitivity to unit-price (inset). ***D***, Average color plots (bottom) and dopamine concentration traces (top) depict dopamine concentration at cues preceding escape with increasing unit-price. Error bars are mean ± SEM.

We used different approaches to analyze dopamine concentration at the avoidance predictive cue versus throughout the safety period. The concentration of dopamine at the presentation of the cue preceding avoidance was defined as the peak concentration ±0.5 s surrounding the event. Avoidance predictive cue-associated dopamine concentrations from individual trials were aggregated, split according to avoidance or escape outcome, and averaged across all animals per unit-price for analysis (Figs. 4*A*,*C*). Voltammograms which contained excessive electrical noise were excluded from this analysis.

As previously described (Oleson et al., 2012), dopamine concentration begins to increase before cue presentation in tasks where the animal can anticipate the timing of its presentation. Because varying the duration of the safety period is known to alter avoidance (Sidman, 1953), and theoretically would alter the valuation of avoidance, we opted to use a fixed, 30-s safety period for the behavioral economics task. As such, it should be noted that the dopamine concentration at the warning signal likely results from both anticipation of the warning signal its actual presentation.

**Figure 5.**
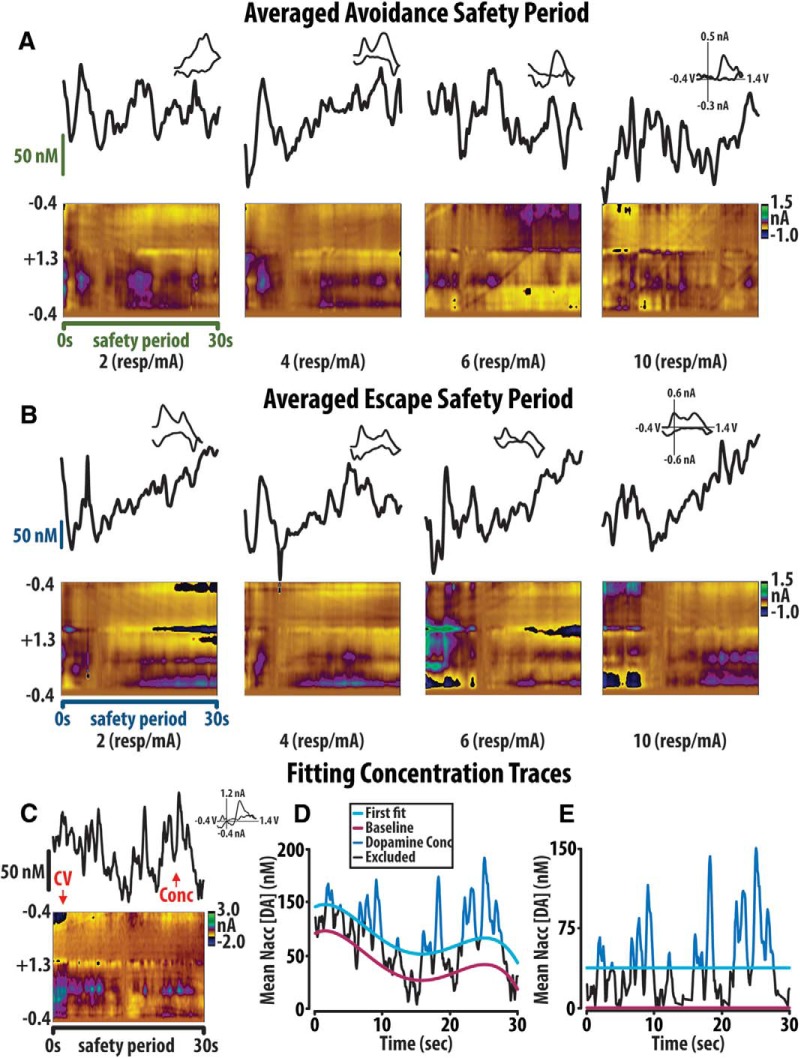
Dopamine during the 30-s safety period following avoidance and escape. ***A***, Averaged color plots (bottom) and dopamine concentration traces (top) limit viable analysis of dopamine transients following successful avoidance and escape (***B***, ***C***). Individual color plot (bottom) and dopamine concentration trace (top) may be fit (***D***) with a baseline polynomic line (red). Addition of a SD to this baseline fit determines the fit (blue). ***E***, The baseline fit is normalized to zero and concentration above this first fit line are considered transients.

**Figure 6.**
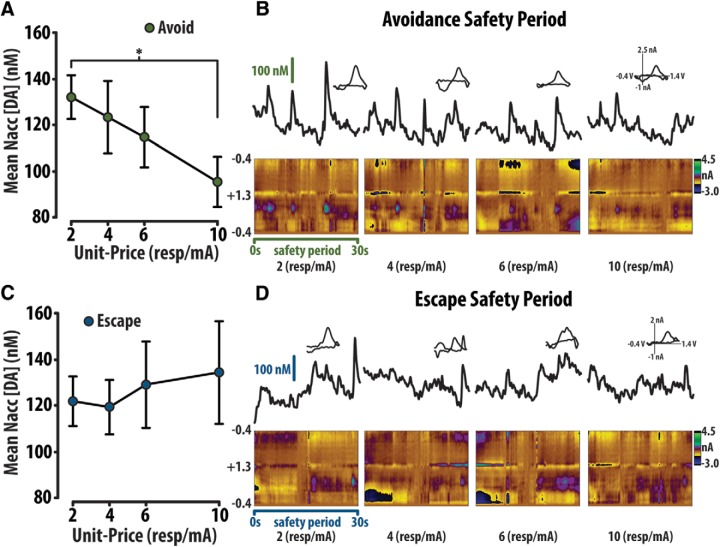
Dopamine during the 30-s safety period following avoidance, but not escape, scales as a function of unit-price. ***A***, The average concentration of accumbal dopamine transients following successful avoidance decreases with increasing unit-price, while the concentration of dopamine following escape did not change as a function of unit-price (***C***). Representative color plots (bottom) and dopamine concentration traces (top) depict these trends at the first four unit-prices following avoidance (***B***) and escape (***D***). Error bars are mean ± SEM.

The pattern of dopamine release during the safety period was distinct as this period is not transient; rather, each safety period lasted 30 s in duration. Thus, to more accurately assess dopamine transient activity during the safety period, rather than analyzing mean dopamine concentration at the onset of the 30-s safety signal we performed an analysis of the amplitude of individual transient events throughout this period. A custom MATLAB program previously described by Schelp et al. (2018) was used to fit a polynomic line to individual 30-s safety period dopamine concentration traces. For each individual safety period trace a polynomic line shift down 1/3 a SD was fit to the dopamine concentration trace as baseline. This SD was then multiplied by 3 and added to the baseline polynomic line to generate a first fit threshold line. Any transients which reached above this threshold line were considered true transients and these concentrations were averaged across each unit-price for both avoidances and escapes (Fig. 5*C–E*). Notably, this program differed from Schelp et al. (2018) through the calculation of SD. Within this program, a region of 2–3 s without dopamine was declared as a baseline to determine the SD of the background signal. Dopamine during the safety associated cue from individual trials were aggregated, split according to avoidance or escape outcome, and averaged across all animals per unit-price for analysis (Fig. 6*A*,*C*). Voltammograms that contained excessive electrical noise were excluded from this analysis.

### Fitting demand curves to avoidance

Demand curves depict the relationship between the consumption of a commodity (in this case avoidance) and changing unit-price. Because demand decreases or decays with increasing price, the desirability of a given commodity may be inferred based on the relative rate of decay of a demand curve. More rapidly decaying demand curves indicate lower demand, or heightened sensitivity to increasing price, while more gradually decaying demand curves indicated greater demand for a given commodity, or reduced sensitivity to increasing price. to generate demand curves depicting avoidance as a function of increasing price. In order to generate demand curves depicting avoidance as a function of increasing price, the avoidance responding observed within the task was fit to an exponentially decaying model though a custom MATLAB script using nonlinear least squares. Within this analysis, total avoidance per unit-price epoch was graphed on the *y*-axis against unit-price on the *x*-axis.

$$Q=\;Q_{\min}+(Q_{\max}-Q_{\min})\;e^{-\alpha C}$$

**Figure 7.**
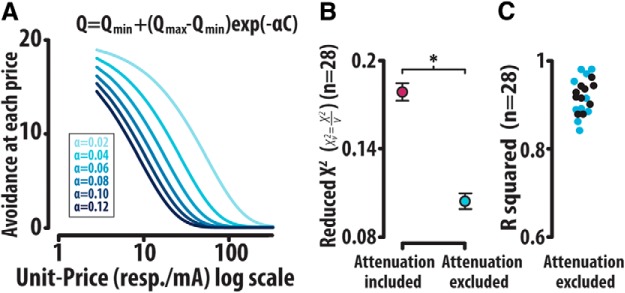
Modeling demand for avoidance. ***A***, An exponentially decaying model fit successful avoidances out of 20 potential cue/response parings as a function of unit-price. Here, the variable α is inversely related to the rate of decay or elasticity of avoidance demand, allowing for a direct assessment of motivation to avoid. ***B***, The attenuation of avoidance at session onset (compare Fig. 3*A*) necessitated the exclusion of the lowest unit-price from economic modeling. The reduced χ^2^ ($X_{v}^{2}$) generated from the exclusion versus inclusion of the lowest unit-price rationalized this exclusion. ***C***, The fits garnered from this equation revealed relatively high *R* ^2^ values (0.92 ± 0.005). Error bars are mean ± SEM.

Analysis revealed that exclusion of unit-price 2 resp/mA, at which attenuated avoidance responding was observed, generated lower $X_{v}^{2}$ values and, therefore, was not included in these fits. Within this equation, the variable α, which describes the rate of decay of the avoidance demand curve, was used to contrast changes in price sensitivity across optogenetic manipulations (Fig. 7).

### Optogenetic manipulation

**Figure 8.**
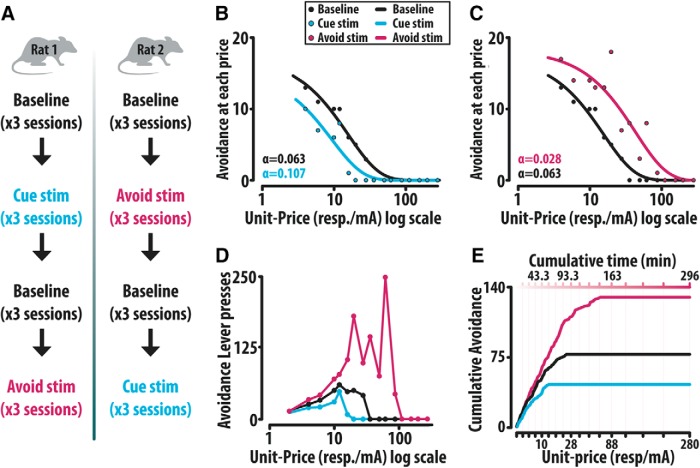
Representative behavior from a single TH-Cre animal. ***A***, Cue and optogenetic augmentation of VTA dopamine cell bodies was counterbalanced across both the TH-Cre and WT groups. Following the establishment of baseline across three behavioral sessions, both groups initially received optical stimulation at either the presentation of the cue (left) or on successful avoidance (right) for three sessions. On completion of the first stimulation paradigm, animals reestablished behavior over three sessions followed by stimulation at the complementary paradigm for three sessions. ***B***, ***C***, Successful avoidances out of 20 potential avoidance opportunities from a representative TH-Cre animal were fit with the exponentially decaying model during cue, avoidance and baseline conditions. ***D***, Avoidance lever responses as a function of unit-price. ***E***, Cumulative avoidance record.

**Figure 9.**
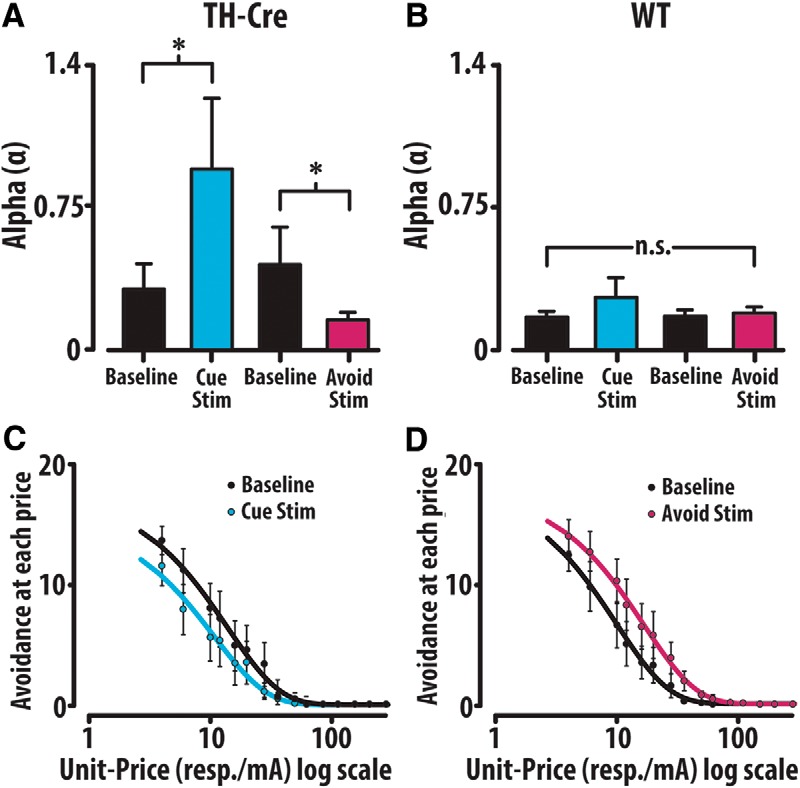
Optogenetic augmentation of VTA dopamine at the cue on avoidance alters the valuation of avoidance. ***A***, α-Values of the TH-Cre group increase with cue stimulation but decrease with stimulation on successful avoidance. ***B***, Cue and avoidance stimulation did not change avoidance performance within the WT control group. ***C***, ***D***, Averaged behavioral trends within the TH-Cre group depict avoidance demand (out of 20 potential avoidance opportunities at each unit-price) with cue and avoid stim relative to baseline. Error bars are mean ± SEM.

To allow sufficient expression of the channelrhodopsin-2 (ChR2) protein, both the transgenic TH-Cre group (*n* = 10) as well as the WT control group (*n* = 10) were given 30 d following surgery before optogenetic stimulation. Both groups received intracranial blue light (473 nm) delivered at 10 pulses, 20 Hz, 0.5-s duration from a laser (opto-engine) controlled by a custom Arduino system (Ng-Evans). Laser output was determined through the Stanford brain transmission calculator to produce a 1mm cone of light of 1 mW/mm^2^ within the brain tissue with a 15-mW output from the ferrule cannula tip and was designed to encompass exclusively the VTA dopamine cell bodies. Stimulation was unilaterally applied to the right hemisphere in all animals. These stimulation parameters have previously been used within the lab and have demonstrated successful augmentation of dopamine within the NAcc (Schelp et al., 2017). Within the economic based avoidance task, rats first established a baseline rate of avoidance across three sessions. Following baseline performance, in a counterbalanced design, rats received optogenetic stimulation for three sessions either at the presentation of the avoidance predictive cue, or when the animal fully met the response requirement and successfully avoided footshock (referred to as “cue” and “avoidance” stimulation, respectively). Following cue or avoidance stimulation, rats reestablished baseline behavior across three sessions, after which the animals received stimulation under the complementary paradigm (cue or avoidance) across three sessions (Fig. 8*A*). Behavior was averaged and contrasted according to baseline, cue, or avoidance paradigm in both the TH-Cre and WT group (Fig. 9).

### Behavioral attenuation

**Figure 10.**
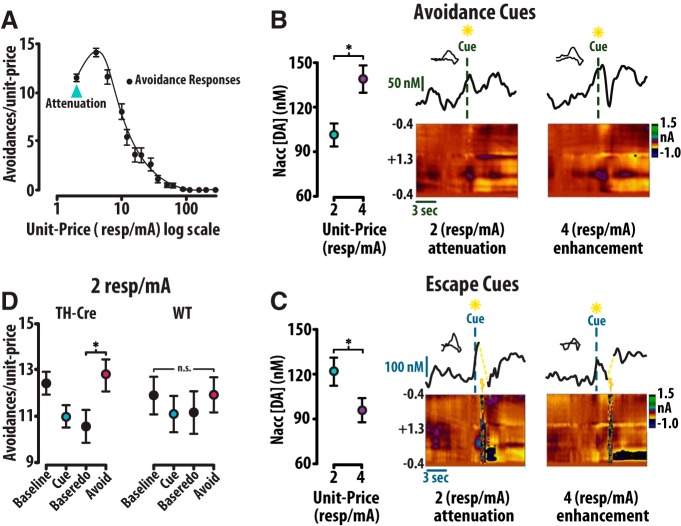
Dopamine scales with avoidance attenuation at session onset and optical augmentation of dopamine attenuation at 2 resp/mA. ***A***, Avoidance at the 2 resp/mA is attenuated relative to avoidance at 4 resp/mA. ***B***, Dopamine concentrations at 2 resp/mA avoidance cues are significantly less than dopamine at 4 resp/mA avoidance cues. Averaged color plots (bottom) with corresponding dopamine concentration traces (top) depict these trends. ***C***, Dopamine concentrations at 2 resp/mA escape cues are significantly less than dopamine at 4 resp/mA escape cues. Averaged color plots (bottom) with corresponding dopamine concentration traces (top) depict these trends. ***D***, Avoidance stimulation, but not cue stimulation, altered avoidance at 2 resp/mA within the TH-Cre group (left), but not within the WT group (right). Error bars are mean ± SEM.

The initial attenuation of behavior (Fig. 10*A*) and the cue associated dopamine concentration at 2 resp/mA were additionally analyzed. Cue associated dopamine concentration were measured for individual trails at both 2 resp/mA and 4 resp/mA. Averaged concentrations at these unit-prices were contrasted for both avoidance and escape outcomes (Fig. 10*B*,*C*). Total avoidance at 2 resp/mA was further analyzed in response to optical stimulation at the cue and upon successful avoidance (Fig. 10*D*).

### Locomotor assessment

**Figure 11.**
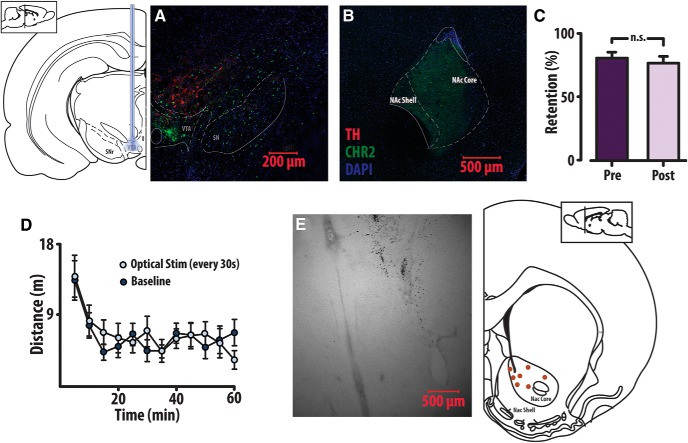
Histology, locomotor assessment, and optical ferrule retention. At the end of optogenetic experimentation, rats were deeply anesthetized with a 50:50 ketamine:xylazine solution and transcardially perfused. Neural tissue was harvested, sliced coronally into 50-µm slices and stained for TH and DAPI. Expression of ChR2 in VTA (***A***) and NAcc (***B***) was verified in all TH-Cre animals. ***C***, Light retention of the optical ferrule cannula was assessed both before and on completion of the experiment. A *t* test revealed no significant difference in ferrule retention. ***D***, To assess for potential locomotor confounds associated with optogenetic stimulation of VTA dopamine neuron cell bodies, rats were first placed in open field chambers and allowed to acclimate for 1 h before moving into either a 1-h stimulation paradigm wherein they received optical stimulation every 30 s, mirroring the max frequency of stimulation animals may receive in the economic task. Baseline and stimulation conditions were counterbalanced across eight animals and did not significantly alter locomotion. ***E***, On completion of the voltammetric experiments, rats were euthanized with CO_2_, and using micromanipulators identical to those used for the voltammetry recordings, stainless steel electrodes were lowered to the same depth as the working electrode during the voltammetry recording. A current to both the stainless-steel electrode and the reference electrode was applied for ∼40 s to lesion the neural tissue at the recording site. Tissue was coronally sectioned into 50-µm slices and placement of the electrodes within the NAcc was assessed. Lesion locations are indicated in red.

To assess for locomotor changes as a result of optogenetic stimulation, transgenic TH-Cre rats (*n* = 8) were placed in locomotor chambers and their movement was tracked using Med-Associates Activity Software. Rats first acclimated to the chambers over the course of 1 h before immediately moving into either a 1-h “stimulation” paradigm wherein they received optogenetic stimulation every 30 s, paralleling the frequency of stimulation animals receive within the economic based avoidance task, or a “baseline” paradigm wherein rats attached to a mock fiber optic patch cable received no stimulation. These conditions were counterbalanced against each other and total distance moved every 5 min was analyzed for both conditions (Fig. 11*D*).

### Histology

On completion of optogenetic experiments, animals were anesthetized with a ketamine xylazine mixture and transcardially perfused with 0.01 M PBS followed by a 4% paraformaldehyde solution. The brains were harvested and rested in a 4% paraformaldehyde solution for 24 h before soaking in a 30% sucrose solution for 48 h, after which the neural tissue was then frozen and kept at −80°C. The frozen neural tissue was sectioned in coronial in 50-µm slices and floated in a 0.01 M PBS/15% normal donkey serum solution for 24 h at 4°C. Tissues was washed with a 0.1% Tween 20/0.01 M PBS for 5 min followed by three 5-min washes of 0.01 M PBS. Tissue was then floated in a 0.1% solution of Immunostar TH primary antibody and 0.01 M PBS for 48 h at 4°C, after which they were washed with Tween 20 and PBS as described above. Following washes, tissue was floated in a 0.1% Alexa Fluor 647 donkey anti-mouse IgG secondary antibody and 0.01 M PBS at 4°C for 12 h, after which the tissue was washed once more with Tween 20 and PBS. Once washed, tissue was mounted on slides with Vector Vectasheild hardset mounting media with DAPI and imaged to identify expression of ChR2, TH, and DAPI in both the NAcc and VTA (Fig. 11*A*,*B*).

On completion of voltammetry experiments, animals were sacrificed using CO_2_. With a micromanipulator, a stainless-steel electrode was lowered to the depth of the recording site and a current was applied to electrically lesion the region. Following lesioning, the brains were extracted, frozen, and stored at −80°C. Tissue was sliced at 50 µm and dry mounted on slides. The mounted tissue was submerged in 95% ethanol/deionized (DI) water for 15 min followed by 1-min submersions in 70% ethanol, 50% ethanol, and two washes in DI water. Tissue was then soaked in Crysal Violet for 1 min, after which it was bathed in two 1-min washes in DI water followed by 15-s washes in 50%, 70%, 95%, and 100% ethanol. This was followed by a ≥8-min wash in Histo-Clear. Once complete, slides were mounted with Paramount and imaged to confirm lesion placement (Fig. 11*E*).

### Statistics

All statistics were performed using SigmaPlot11. First, Shapiro–Wilk was used to assess for normality and Brown–Forsythe was used to assess for equal variance. If these tests passed, ANOVA were used; if these tests failed, equivalent non-parametric statistics were used.

## Results

### Relationship between alpha and price elasticity of demand

Demand curves are a common tool used by economists to measure price sensitivity. Demand curves depict the relationship between consumption of a commodity (in this case the avoidance of harm) and price. Demand curves usually show a negative gradient (i.e., the law of demand), where consumption decreases with increasing price. The rate at which the negative slope decays can be used to make inferences regarding the value individuals place on the commodity being consumed. When demand curves decay at a faster rate they are said to be more elastic. Price elasticity of demand is defined as the change in the quantity of the commodity being consumed in response to an increase in price. In the present study, the number of successful avoidance responses at each price represents the quantity of the demanded commodity. We measure the elasticity of demand by computing the variable α, which represents the cost at which the elasticity of demand is exactly −1, meaning consumption drops by one percentage in response to a one percentage increase in price. A higher α-value indicates the demand for the good is more elastic, suggesting the value of the commodity is diminished. In contrast, a lower α-value indicates the demand for the good is relatively inelastic, suggesting the value of the commodity is enhanced.

### A behavioral economics task was used to investigate the role of dopamine in the valuation of avoidance

To assess the role of mesocorticolimbic dopamine release events in the valuation of avoidance, we used a behavioral economic-based shock avoidance task. Our approach both builds on previous within session designs that characterized the demand for sucrose and cocaine (Oleson and Roberts, 2009; Schelp et al., 2017) as well as expands on a recent between sessions approach that was the first to apply behavioral economics to negative reinforcement (Fragale et al., 2017). As a within session design is more conducive to neural monitoring, we began by exploring the effects of increasing the unit-price (response requirement/mA shock avoided) of avoidance within individual sessions. We first sought to increase unit-price by manipulating the mA shock avoided (1.0–0.13 mA) across fixed epochs. To accomplish this, a range of six unit-prices were presented in descending order (Fig. 1*A*) and avoidance was analyzed as a function of unit-price. Avoidance failed to consistently scale with price when price was manipulated by changing shock amplitude across within session epochs. Next, we sought to manipulate unit-price by increasing the response requirement to avoid across within session epochs. As would be predicted by the law of demand, this task engendered a stable pattern of behavior hallmarked by a negative price elasticity, meaning that the demand to avoid decreased with increasing price (Fig. 1*B*). However, we also observed an initial attenuation of behavior at session onset which is distinct from previous reports investigating the demand to obtain sucrose (Schelp et al., 2017) or cocaine (Oleson and Roberts, 2009). To address order effects, we attempted to randomize the order in which the response requirements were presented (Fig 1*C*, upper). As was previously reported for sucrose demand (Schelp et al., 2017), animals showed aberrant response patterns with randomization of unit-price (lower). Thus, we proceeded with a task in which the unit-price was manipulated by increasing response requirement and presented prices in a consistent ascending order. Finally, to ensure that all animals reached a price at which they failed to sustain demand, the total number of unit-prices was increased from 6 to 16.

Within the selected ascending response requirement task, a compound cue signaled the opportunity to respond on a lever in order avoid an unchanging electrical footshock (0.5 s, 0.5 mA). Failure to meet the response requirement within a set timeframe resulted in recurrent footshock until the full response requirement was met. This outcome is defined as escape. Successfully meeting the response requirement within a set timeframe resulted in a safety period signaled by a tone without the occurrence of footshock. This outcome is defined as avoidance. Following either avoidance or escape, a tone accompanied by house light illumination signaled a 30-s safety period (Fig. 2*A*). The end of each safety period coincided with the onset of the next trial, starting with presentation of the avoidance predictive cue. On acquisition of avoidance under a FR1 reinforcement schedule, animals were introduced to unit-price manipulation through an increase in response requirement across discrete, time-based epochs in each daily session. Rats were initially trained to perform multiple lever press responding across six unit-prices (Fig. 2*B*). Unit-price epoch duration was modulated to allow for a maximum of 20 avoidance opportunities within each unit-price (column 4) and additional time was provided to rats to meet increasing response requirement (column 3). On acquisition of multiple lever press responding rats were placed in an economic based task with 16 ascending unit-prices (Fig. 2*C*). Once animals were successfully trained to escape, then avoid at ≥50% avoidance on an FR1 schedule, animals took an average of 13.9 ± 3.1 sessions to acquire multiple lever press responding and 19.5 ± 2.9 sessions within the economic task to establish stable baseline behavior (Fig. 2*D*). On establishment of baseline behavior, animals entered optogenetic and FSCV experimentation.

While data were primarily analyzed in terms of successful avoidance outcomes at each unit-price, note that the additional delay required to meet increasing response requirements adds a conceptually important opportunity cost (cf. Fig. 2*B*,*C*, columns 2 and 3). Thus, total cost to the animal results from both effort and opportunity costs.

### Unique avoidance demand curves were generated to measure price sensitivity

This task allowed us to generate within session demand curves by plotting total avoidance responses per epoch against unit-price. Unique demand curves emerged over the course of acquisition of the economic task, with an attenuation of avoidance at 2 resp/mA becoming apparent in experienced animals (Fig. 3*A*). The ratio of total avoidance at 4 versus 2 resp/mA significantly increased over the course of training (one-way RM ANOVA: *F*_(2,44)_ = 9.87, *p* < 0.001; Tukey *post hoc*: start of training vs end of training *p* < 0.001; Fig. 3*B*). Similar economic-based food-seeking tasks do not demonstrate this attenuation at session onset (Schelp et al., 2017; Fig. 3*C*), suggesting that additional inhibitory neural circuitry might be recruited during economic assessments of avoidance (Jhou et al., 2009). The ratio of avoidance 4 resp/mA versus attenuated avoidance at 2 resp/mA in both a transgenic TH-Cre and WT group develops and stabilizes over the course of training (Fig. 3*D*). While a measure of demand, α, similarly stabilizes over the course of training in both the TH-Cre and WT group (Fig. 3*E*), suggesting this attenuation might be a learned suppression that develops across repeated sessions, each of which terminates when the animal receives 15–20 consecutive footshocks.

### Dopamine release events correspond to value associative cues in avoidance

To evaluate the role of NAcc dopamine during the valuation of avoidance, we employed FSCV to measure transient changes in dopamine concentration time locked (±0.5 s) to associative cues in the behavioral economics-based task. We exclusively analyzed dopamine concentration within the second through fourth unit-price because, 100% of animals maintained responding in this range and a concurrent attenuation in behavior and dopamine concentration was observed at the first price point. We independently address these attenuated responses later in the manuscript. The nA changes in current were converted to nM concentration by using PCR and lab-specific computational factors (see Materials and Methods). Dopamine concentration files were arranged around one of two events: (1) avoidance predictive cue and (2) safety associated cue. Dopamine at the avoidance predictive cue was quantified and analyzed as a group mean because the mean data were representative of individual trials (cf. Fig. 4*A*, inset, vs *B*,*D*). Dopamine during the safety period was quantified and analyzed at the level of the individual transient because mean data were not representative of individual trial (cf. Fig. 5*C* vs 5*A*,*B*).

### Dopamine at the avoidance predictive cue

In successful avoidance trials we found that dopamine concentration at the avoidance predictive cue scaled inversely to unit-price [one-way repeated measures (RM) ANOVA: *F*_(2,20)_ = 29.507, *p* < 0.001; Tukey *post hoc*: unit-price 4 vs 6 *p* < 0.001, 4 vs 10 *p* < 0.001; Fig. 4*A*]. A representative avoidance trial (Fig. 4*A*, inset) demonstrates individual trial similarity to mean dopamine concentration plots for all animals. Average color plots demonstrate these avoidance cue trends at each unit-price (Fig. 4*B*, bottom) with corresponding concentration traces (Fig. 4*B*, top). Contrary to our predictions (Oleson et al., 2012), in escape trials we found that dopamine concentration also showed an inverse relationship to unit-price (one-way RM ANOVA: *F*_(2,20)_ = 3.916, *p* = 0.049; Tukey *post hoc*: unit-price 4 vs 10 *p* = 0.040; Fig. 4*C*). Within escape trials, dopamine concentration did not, however, demonstrate sensitivity to number of shocks subsequently received (one-way RM ANOVA: *F*_(3,23)_ = 2.14; *p* = 0.14; Fig. 4*C*, inset). As would be predicted (Oleson et al., 2012), an increase in dopamine concentration was observed before cue presentation. Given that the safety period duration preceding cue presentation is consistent (30 s) is it possible that this preemptive increase in dopamine reflects anticipation of the cue. As such, it should be noted that the dopamine concentration at the warning signal likely results from both anticipation of the warning signal its actual presentation. However, varying the duration of the safety period is known to alter avoidance (Sidman, 1953), and theoretically would alter the valuation of avoidance, thus it is necessary to use a fixed, 30-s safety period for the behavioral economics task. Together, these data demonstrate that, as in reward seeking, dopamine concentration at an avoidance predictive cue generally scales with price.

### Dopamine at the safety-associated cue

We further analyzed the amplitude of dopamine transient events during the 30-s safety period following both avoidance and escape outcomes. Unlike dopamine concentration transients time locked to cue presentation, averaged safety period color plots (bottom) and corresponding concentration traces (top) following avoidance (Fig. 5*A*) and escape (Fig. 5*B*) are not representative of individual safety period trials (Fig. 5*C*). Therefore, to analyze safety period transients, dopamine concentration traces from individual trials were fit with a baseline polynomial line (Fig. 5*D*, red) and a SD was added to the baseline fit to generate a cutoff (Fig. 5*D*, blue). The baseline was set to zero and concentrations above the first fit line were analyzed as transients (Fig. 5*E*). Analysis of individual safety period trials revealed an inverse relationship between average transient amplitude and unit-price following successful avoidance (one-way RM ANOVA: *F*_(3,27)_ = 3.50, *p* = 0.037; Tukey *post hoc*: unit-price 2 vs 10 *p* = 0.029; Fig. 6*A*). This trend is demonstrated with representative color plots (Fig. 6*B*, bottom) and corresponding concentration traces (Fig. 6*B*, top). Conversely, concentration directly following escape demonstrated no relationship to unit-price (one-way RM ANOVA: *F*_(3,27)_ = 0.902; *p* = 0.460; Fig. 6*C*,*D*). These data suggest that dopamine only represents the valuation of avoidance during the safety period following successful avoidance.

### Modeling the elasticity of demand in avoidance

To measure changes in price sensitivity, total avoidance versus unit-price was fit with an exponentially decaying model:

$$Q=\;Q_{\min}+(Q_{\max}-Q_{\min})\;e^{-\alpha C}$$

with the variable α describing the rate of decay, $Q_{max}$ depicting avoidance at zero price, $Q_{min}$ depicting minimum avoidance, and *C* representing unit-price. Notably, α is inversely related to avoidance performance, with lower α values signifying higher avoidance while larger α values reflect lower avoidance (Fig. 7*A*). As previously noted (Fig. 3*A*), an initial attenuation in demand occurs at 2 resp/mA. Similarly to the loading phase of cocaine self-administration (Oleson et al., 2011; Bentzley et al., 2014), this initial attenuation may be influenced by additional variables other than price. Thus, we investigated whether removing the first data point resulted in better-fitted demand profiles. To account for differences in the degrees of freedom between fitting 16 and 15 unit-prices, the reduced χ^2^ ($X_{v}^{2}$) was calculated with both the inclusion and exclusion of the lowest unit-price. It was revealed that the exclusion of the lowest unit-price yielded significantly lower $X_{v}^{2}$ (included: 0.178 ± 0.0059; excluded: 0.104 ± 0.0053; Mann–Whitney rank sum test: U_(n1=n2=272)_ = 20155.0, *p* < 0.001; Fig. 7*B*) as well as increased *R* ^2^ values (included: 0.85 ± 0.005; excluded: 0.91 ± 0.005; Fig. 7*C*). Given that removal of the first data point results in significantly lower $X_{v}^{2}$, we opted to analyze demand profiles generated in the avoidance task after removing the first data point, as is done when analyzing demand profiles of cocaine self-administration (Oleson et al., 2011). This approach, combined with optogenetics, allowed us to investigate how dopamine neurons causally modify economic demand in avoidance.

### Dopamine release events causally modify demand for avoidance

Optogenetics can be used to assess the causal relationship between patterns of neural activity and behavior (Steinberg et al., 2013). Thus, we next sought to investigate how optically increasing dopamine release through selective optogenetic activation (10 pulses, 20 Hz, 0.5-s duration) of ChR2 expressing dopamine neurons of the VTA alters demand for avoidance. While future studies are necessary to parse the role of dopamine release at its various terminal projections, we opted to begin by augmenting mesoscorticolimbic dopamine release at its origin. Initially, animals from both experimental TH-Cre and WT control groups were trained to respond on a lever to terminate and escape footshock. On acquisition of escape behavior animals were moved into an FR1 avoidance task until >50% avoidance was reached and maintained. Subsequent to the establishment of >50% avoidance behavior, animals were trained to respond multiple times on the lever to avoid footshock across six unit-prices. On acquisition of multiple lever presses, animals were trained on the economic based shock avoidance task until a baseline rate of avoidance was established across three sessions. After establishing stable baseline behavior, we provided unilateral optogenetic stimulation of the VTA cell bodies of the right hemisphere at either the presentation of the avoidance predictive cue or on successful avoidance. All animals were tested under both stimulation conditions. We investigated the effects of optical stimulation at the first event for three sessions followed by reestablishment of baseline behavior and then stimulation under the complimentary event. The order of stimulation (avoidance predictive cue vs successful avoidance) was counterbalanced within both the TH-Cre group and the WT group (Fig. 8*A*). The number of successful avoidances out of 20 avoidance opportunities within each unit-price was fit with an exponentially decaying model (Fig. 7*A*) and optogenetic-induced changes in demand were assessed by comparing α values to baseline values. Representative cumulative response records, response-price curves and corresponding demand curves from a single TH-Cre animal depict the resulting patterns of behavior across all stimulation conditions (Fig. 8*B–E*).

Analysis of the TH-Cre group revealed that stimulation at the avoidance predictive cue significantly increased α (decreased demand to avoid) while stimulation at successful avoidance significantly decreased α (increased demand to avoid). Cue associated baseline and avoidance associated baseline were not significantly different (Friedman RM ANOVA on ranks: X^2^_(3)_ = 17.88, *p* < 0.001; Tukey *post hoc*: baseline-1 vs cue *p* = 0.017, baseline-2 vs avoid *p* = 0.017, cue vs avoid *p* = 0.003, baseline-1 vs baseline-2 *p* = 0.072; Fig. 9*A*). Conversely, the WT group demonstrated no change in α value with optogenetic stimulation (one-way RM ANOVA: X^2^_(3)_ = 1.08, *p* = 0.78; Fig. 9*B*). Averaged avoidance within the TH-Cre group depicts changes in demand with cue (Fig. 9*C*) and avoidance (Fig 9*D*) stimulation. Neither stimulation condition produced a significant change in avoidance or escape response latency when compared to baseline (one-way RM ANOVA: Cre avoidance: *F*_(3,39)_ = 0.824, *p* = 0.49; Cre escape *F*_(3,39)_ = 2.87, *p* = 0.055; Friedman RM ANOVA on ranks: WT avoidance: X^2^_(3)_ = 1.2, *p* = 0.75; WT escape: X^2^_(3)_ = 2.0, *p* = 0.56). As in reward seeking (Schelp et al., 2017), both $Q_{min}$ and $Q_{max}$ demonstrated no significant change in response to optogenetic manipulations, suggesting that α is more highly associated with changes in avoidance price sensitivity. These data demonstrate that optical stimulation of dopamine neurons influences the demand to avoid electrical footshock, supporting the notion that dopamine causally modifies the valuation of avoidance.

### Dopamine is concurrently attenuated with avoidance at session onset

We additionally sought to analyze the attenuation of avoidance at 2 resp/mA (Fig. 10*A*). Dopamine time locked to cues (±0.5 s) preceding avoidance at 2 resp/mA is significantly lower than cue evoked dopamine at 4 resp/mA (Fig. 10*B*). Averaged color plots (bottom) and dopamine concentration traces (top) depict these trends. Conversely, dopamine time locked (±0.5 s) to cues preceding escape at 2 resp/mA was greater than at 4 resp/mA (Fig. 10*C*). Averaged color plots (bottom) and dopamine concentration traces (top) demonstrate this trend. Optical stimulation of dopamine neurons similarly influences the attenuation in demand observed as session onset. In the TH-Cre group, optical stimulation at successful avoidance, but not at the avoidance predictive cue, reduced attenuation relative to baseline performance (one-way RM ANOVA: *F*_(3,39)_ = 3.76, *p* = 0.022; Tukey *post hoc*: baseline-1 vs cue *p* = 0.28, baseline-2 vs avoid *p* = 0.042). Conversely, the WT group demonstrated no change in attenuated avoidance in response to optical stimulation (one-way RM ANOVA: *F*_(3,39)_ = 1.00, *p* = 0.41; Fig. 10*D*). Our electrochemistry data demonstrate that dopamine at the avoidance predictive cue is concurrently attenuated with behavior at session onset in this task. We believe this attenuation at session onset is a learned suppression that develops following repeated training sessions in which sessions terminate with repeated electrical footshock. Our optogenetics data reveal that dopamine release is sufficient to rectify attenuation of avoidance demand at session onset. Together, these observations support the view that heightened dopamine release can overcome the effects of negative affect on behavior (Chaudhury et al., 2013; Tye et al., 2013).

### Additional experimental controls and considerations

On completion of optogenetic experiments, neural tissue was harvested and expression of ChR2 in the VTA and NAcc was confirmed in all TH-Cre animals (Fig. 11*A*,*B*). Optical ferrule cannulae, which were assessed before and after surgical implantation, demonstrated no significant decrease in retention (paired two-tailed *t* test: *t*_(7)_ = 1.59, *p* = 0.15; Fig. 11*C*). to address potential locomotor confounds produced by optical stimulation, recurrent optogenetic stimulation of VTA dopamine neurons was conducted within the TH-Cre group during an open field test. This augmentation failed to increase horizontal activity relative to baseline (paired two-tailed *t* test: *t*_(7)_ = −0.363, *p* = 0.727), suggesting changes in overall activity played a minimal role in our demand profiles. On completion of the voltammetric experiments, animals were sacrificed and electric lesions at the depth of the recording site confirmed electrode placement within the NAcc (Fig. 11*E*).

## Discussion

We investigated the role of mesocorticolimbic dopamine release events in the valuation of avoidance using a combination of behavioral economics, modeling, electrochemistry, and optogenetics. After an initial attenuation of dopamine release and behavior at session onset, accumbal dopamine concentration scaled inversely with price during the active avoidance of signaled footshock. Dopamine at the warning signal decreased with price irrespective of the outcome (avoidance vs escape); however, dopamine only decreased with price at the safety associated cue following successful avoidance. We interpret these distinct outcome-specific responses to suggest that dopamine concentration before the behavioral action was predictive of value, whereas dopamine concentration following action was reflective of the outcomes value.

We next sought to assess the causal role of dopamine in the valuation of avoidance within an economic framework. A central principle of economics is that as the price of a commodity increases, its demand decreases. This principle is commonly demonstrated using demand curves, in which consumption of a commodity is plotted against price. According to the law of demand, consumption typically decreases with increasing price. The resulting negative gradient represents the elasticity of demand, or how sensitive consumption of the commodity is to increasing price. In the present study, consumption is defined as the number of successful avoidance responses at each price (responses/mA). If avoidance became more sensitive to price, the resulting demand curve would decay at a faster rate; if avoidance became less sensitive to price, the resulting demand curve would decay at a slower rate. We can then make inferences regarding the value of avoidance by measuring the rate of decay. We compute the rate at which demand curves decay by solving for the variable α, which represents the cost at which the elasticity of demand is exactly −1. At this point, consumption drops by one percentage in response to a one percentage increase in price. A higher α-value indicates the demand for the good is more elastic, suggesting the value of the commodity is diminished. In contrast, a lower α-value indicates the demand for the good is relatively inelastic, suggesting the value of the commodity is enhanced. In accordance with our previous work assessing the role of dopamine in the valuation of a sugar reward (Schelp et al., 2017), we predicted that increasing dopamine release at an avoidance predictive cue would decrease α, whereas increasing release at successful avoidance would increase α.

Optically stimulating dopamine cell bodies in the VTA at an avoidance predictive cue rendered animals more sensitive to price (i.e., α increased), consistent with a negative reward prediction error. We infer that heightened release at cue presentation signaled a beneficial outcome, a prediction that was then violated by the occurrence of footshock outcomes of the same amplitude. Optically increasing release at successful avoidance made animals less sensitive to price (i.e., α decreased), consistent with a positive reward prediction error. We infer that heightened release at successful avoidance signaled the outcome was better than predicted, indicating a good value worth seeking. Our data build on the notion that transient dopamine release events can represent subjective value (Schelp et al., 2017; Schultz et al., 2017) and further clarify that these value signals not only represent the value of pursuing reward, but also the value of avoiding harm.

The mesocorticolimbic pathway originates from dopamine neurons in the VTA and projects to motivational circuitry throughout the brain, most prominently the NAcc of the basal ganglia. The NAcc is thought to integrate transient dopamine signals with an array of converging neural input to generate goal directed actions via the basal ganglia (Haber, 2014; Jin et al., 2014; Floresco, 2015; Graybiel and Grafton, 2015). Both the pursuit of reward (positive reinforcement) and the active avoidance of harm (negative reinforcement) require an increase in action to ultimately promote behavioral fitness and survival. While it is likely that individual dopamine neurons heterogeneously represent rewarding and aversive stimuli (Lammel et al., 2014; Pignatelli and Bonci, 2015), our data suggest that the summation of dopamine neural output is increased in the NAcc during active avoidance. Overall, we believe that these transient release events act on the basal ganglia to strengthen action sequences directed toward optimal outcomes. However, it is important to note that optical stimulation of the VTA dopamine neuron cell bodies leads to increases in concentration in various neural substrates implicated in avoidance (Darvas et al., 2011; Pignatelli and Bonci, 2015). Future studies are needed to investigate the specific role that dopamine plays in the valuation of negative reinforcement in various regions including the frontal cortex, amygdala, and striatum.

It is important to note that there are distinct forms of avoidance and dopamine may play unique roles in each. For example, if transient dopamine release events are significant for action generation, then dopamine release should be suppressed in passive avoidance, a situation where animals must inhibit action to avoid a negative outcome (Ögren and Stiedl, 2010). Unsignaled “Sidman” avoidance is another interesting consideration. In Sidman avoidance animals show active avoidance despite the absence of an exteroceptive warning signal (Sidman, 1953). It is possible that during unsignaled avoidance the lever itself, in addition to proprioceptive associations that develop during the lever response, function as conditioned stimuli. Another possibility is that dopamine may guide unsignaled avoidance through representation of interoceptive timing cues (Oleson et al., 2014; van Rijn et al., 2014). Indeed, as previously noted (Fig. 4*B*,*D*), we observed an increase in dopamine concentration preceding the cue, implying an anticipation of cue presentation as animals perform within this task. While additional studies are required to fully understand how dopamine is related to the various forms of avoidance, it is important to note that distinct patterns of dopamine release likely distinguish them.

In contrast to active avoidance, transient dopamine release is suppressed by unavoidable aversive events and their associative stimuli. Using FSCV, the Roitman group has demonstrated that a variety of aversive stimuli transiently decrease accumbal dopamine concentration (Roitman et al., 2008; Ebner et al., 2010; McCutcheon et al., 2012; Fortin et al., 2016). Similarly, stimuli associated with inescapable electrical footshock suppress accumbal dopamine concentration within the NAc (Badrinarayan et al., 2012; Oleson et al., 2012). While these dopamine release events likely represent the summation of VTA dopamine neural activity, there is growing evidence to support heterogeneous representation of rewarding and aversive stimuli at the level of the single unit (Lammel et al., 2012; Pignatelli and Bonci, 2015). While the majority of electrophysiology studies report that dopamine neurons are inhibited by aversive stimuli (Mileykovskiy and Morales, 2011; Tan et al., 2012), numerous reports of excitations exist as well (Brischoux et al., 2009; Matsumoto and Hikosaka, 2009). While some of these reports were likely confounded by the inclusion of non-dopamine neurons (Ungless and Grace, 2012), it is likely that many factors influence whether individual neurons are excited or inhibited by an aversive event, including the subpopulation of neurons surrounding the recording site (Lammel et al., 2012) and the behavioral context in which aversion is introduced (Matsumoto et al., 2016).

Dopamine responses in active avoidance further depend on the behavioral history of the subject and the behavioral context in which avoidance is assessed. Notably, our observations here are discrepant from a previous FSCV characterization of dopamine in signaled active avoidance (Oleson et al., 2012), which observed distinct responses at the avoidance predictive cue during the escape outcome. In the present study, dopamine scaled with price at avoidance predictive cues, even in escape. Conversely, Oleson et al. (2012) reported that dopamine was inhibited at the avoidance predictive cues preceding escape. We believe that this discrepancy is due to a combination of behavioral history and behavioral context. While Oleson et al. (2012) observed a suppression in dopamine concentration and behavior when the avoidance predictive cue resulted in escape rather than avoidance, animals were simply trained to avoid footshock under an FR1 schedule in up to 50% of trials and sessions terminated after a fixed amount of time. In the current task, animals were extensively trained to avoid in several different paradigms (see methods) before being tested in the much more strenuous behavioral economics task. Furthermore, in the current task, sessions only terminate after 15 shocks or 20 escapes occur in a consecutive order. Thus, we believe that this initial attenuation of both behavior and dopamine release is induced by session onset, which comes to predict a negative session end. This powerful negative prediction develops and stabilizes over the course of economic avoidance training (Fig. 3*D*,*E*), supporting the notion that it is a learned association and distinct from changes in performance with optical stimulation. Once the animals had established stable baseline performance in the behavioral economics task, the negative prediction represented by session onset was sufficient to attenuate dopamine release and active avoidance. Optically augmenting dopamine release at avoidance rectified this behavioral attenuation, further supporting recent evidence that heightened dopamine release can overcome the effects of negative affect on behavior (Chaudhury et al., 2013; Tye et al., 2013).

Dopamine responses at the avoidance predictive cue depend on the behavioral outcome (avoidance vs escape). We observed distinct responses to avoidance associative cues depending on whether animals avoided or escaped footshock. During successful avoidance, cue-evoked dopamine concentration scaled with changing price, resulting from both an increase in effort and opportunity costs, and corresponded to the avoidance outcome. During escape outcomes, cue-evoked dopamine concentration scaled with price irrespective of behavior. We interpret the distinct responses at the avoidance predictive cue to suggest that dopamine is continually representing avoidance value but is being simultaneously attenuated with behavior at session onset. We surmise this concurrent attenuation at session onset results from afferent inhibitory input, possibly from the lateral habenula via the rostromedial tegmentum (Jhou et al., 2009).

Dopamine responses during the safety period also depend on the behavioral outcome. We observed dopamine concentration scaled with price during the signaled safety period when animals successfully avoided footshock, but not following escape. The distinct dopamine responses observed during the safety period may be relevant in the context of avoidance learning. Successfully avoiding an aversive event was the optimal outcome under our experimental conditions. Thus, dopamine might exclusively convey value during the safety period after optimal outcomes, thereby promoting their recurrence. While entering safety after escape is a beneficial outcome as well, dopamine may fail to represent the value of this less optimal outcome. Rather, we reason that dopamine generally represents the safety signal proceeding escape as a positive relief from pain (Navratilova and Porreca, 2014).

Avoidance outcomes produce unique behavioral and neurochemical responses when compared to rewarding outcomes (Schelp et al., 2017), suggesting that avoidance is more than simply reward redux. While the avoidance of an aversive event is rewarding, the avoidance outcome still carries aversive qualities. When animals run toward a goal box containing a footshock-associated rewarding outcome they show an approach-avoidance conflict, wherein retreat from the goal box accompanies the pursuit of reward (Geist and Ettenberg, 1997). In addition, unique demand profiles and dopamine responses are observed during the valuation of avoidance and reward (Roitman et al., 2008). The aversive attributes of footshock avoidance likely recruit additional neural circuits that interact with the mesocortiolimbic pathway during negative reinforcement. Thus, while mesocorticolimbic dopamine release events similarly represent the value of both rewarding and avoidance outcomes, we surmise that the overall neural circuitry underlying positive and negative reinforcement is distinct.

In conclusion, we demonstrate that accumbal transient dopamine release events scale proportionally to the value of avoidance outcomes and capably modify the valuation of active avoidance. Our data refute the notion that mesocorticolimbic dopamine is exclusively involved in positive reinforcement (Fiorillo 2013) and, rather, indicate that dopamine represents the value of all advantageous outcomes, including the avoidance of harm.

## Synthesis

Reviewing Editor: Carmen Sandi, Swiss Federal Institute of Technology Decisions are customarily a result of the Reviewing Editor and the peer reviewers coming together and discussing their recommendations until a consensus is reached. When revisions are invited, a fact-based synthesis statement explaining their decision and outlining what is needed to prepare a revision will be listed below. The following reviewer(s) agreed to reveal their identity: Armin Lak, Wolfram Schultz

Using experimental economics in the neuroscience of reward and decision-making (including negative value = punishment) is likely to reveal many novel and important aspects. This is an important study that follows in the footsteps of their preceding PNAS paper.

However, the reviewers and editor have identified important issues that need to be addressed, some of them related to figures numbering, citations and, particularly, to the writing style. See specific comments below:

1. Figures: There are no numbers on the figures - they should be included and well cited in the text.

2. Citations: In a few places in the manuscript, the citations could be more accurate. Currently, some of the citations are not reflecting the results and conclusions of the cited paper. I will point out one, and I would like to invite authors to carefully evaluate the accuracy of each citation. Here is one instance:

Line 92-3: It was then shown that the extent of the dopamine response corresponds to predicted reward magnitude (Bayer and Glimcher, 2005, ...)

Bayer and Glimcher, 2005 explicitly states that they found no such relation. From their paper: “We found no evidence that this signal predicted the magnitude of the upcoming reward, a fact which likely reflects the temporal uncertainties of our particular task (Schultz et al., 1997).”

3. Writing style: A critical limiting factor of this article is the serious deficits in the writing style. The descriptions are obscure, there are insufficient explanations (as if the authors had not understood their study, although I am sure they did), and the whole text is un-understandable for both the specialist and the non-specialist, both for economics and neuroscience.

The authors can use the comments below regarding the Results sections as guidelines for improving also the rest of the paper (note that specific comments on those other sections are not provided, even though careful revision of those is also needed). Before submitting a revision, the authors should make sure that the new version is understandable and following very good scientific writing standards.

Abstract:

A small detail: To make a paper on both reward and punishment more understandable, the term ‘prediction error’ without indicating ‘reward’ or ‘punishment’ is unclear: the terms positive or negative prediction error should be replaced by positive or negative reward prediction error, or by positive or negative punishment prediction error, depending on what you mean.

After the considerations stated below, I might understand the sentence “Increasing release at avoidance made animals less sensitive to price, consistent with a positive prediction error” (lines 69-70) as saying that optogenetic dopamine stimulation adds positive value to whatever happens at that moment, which may well reduce the negative value of price (and thus make animals ‘less sensitive to price’). Is that correct? But I don't understand what the sentence means “Increasing release at an avoidance predictive cue made animals more sensitive to price, consistent with a negative prediction error” (lines 69-70). Would the negative PE be at the cue, or at the avoidance? In the latter case, optogenetically, artificially increased dopamine would generate enhanced value at the cue, which then could lead to a negative PE at the not-value-enhanced avoidance. Is that meant? Please say so.

Introduction: The part concerning dopamine and avoidance is made unnecessarily complicated and unclear. The dopamine release with avoidance may well reflect the relief derived from the avoidance, and behavioral studies have shown that this relief has positive reinforcing properties. Thus, a dopamine increase with avoidance, or even the prediction of avoidance, may be a simple positive reinforcement signal. That function would not be properly captured by the general wording of ‘a general role for dopamine in avoidance’ (line 106). Phrased more precisely would make the message of the paper much clearer. Of course, that argument may reflect only one of several possible interpretations, and if there are alternative reasonable interpretations, they should be stated clearly as well.

The wording on lines 121-123 is entirely un-understandable: what does it mean in terms of reward value if “dopamine release at avoidance-related cues should scale with avoidance costs”? Should the dopamine release go up or down with increasing cost (scaled positively or negatively; dopamine should scale negatively with cost=negative reward)? Just say it, and then say clearly what this directional change would mean in terms of dopamine value coding. Maybe you could explain this in terms of “worth an animal places on an outcome” mentioned above. Once we understand this sentence, we can search where the reward prediction error comes in and in which direction it should go. Further, the sentence starting at line 124 is perfectly un-understandable.

Maybe it would help if the authors could define / explain at the outset whether price and demand have positive or negative reward value, each one separately. Price (= effort, energy expenditure, like lever pressing) should be cost (and dopamine concentration goes down accordingy (fig 4), but what about demand? With increasing price, theoretically demand should drop, so they are inversely related (although this relationship was found only in parts of the behavioral data). Would then ‘increasing the demand to avoid’ be indicative of a price drop = increased value? This is a huge step, with plenty of assumptions, and not taking it apart and describing it well does not help. In fact, I could not find anywhere a definition of how demand was measured: was it the avoidance / unit-price shown in fig 1? It should be the number of avoided shocks, or the intensity of the avoidance in terms of lever presses to be compatible with standard definitions for demand (which one did you measure? Please state at beginning of Results section). Wikipedia says for demand “the amount that consumers are willing and able to purchase” - such a statement should be repeatedly made throughout the paper (and we should not need to go to Wikipedia when reading a paper).

What does avoidance / unit-price stand for: is this a ratio as the ‘/’ symbol indicates: avoidance divided by unit-price (?), or does this mean simply avoidance at each unit-price (which would be correct)? The text (line 388) seems to say the latter, but the y-axis label in fig 3a would then be misleading.

It would be helpful if the authors could describe what fig 3a shows. I think Reviewer 1 got it wrong when stating “rats avoid less for lowest avoidance price compared to the second price”, as this would violate the law of price and demand, as shown in fig 3a (the lower the price, the higher the demand). A succinct description could help before going into sidelines about learning effects.

## Author Response

Synthesis Statement for Author (Required):

Using experimental economics in the neuroscience of reward and decision-making (including negative value = punishment) is likely to reveal many novel and important aspects. This is an important study that follows in the footsteps of their preceding PNAS paper.

We thank the reviewers and editor and believe our manuscript is much improved after addressing your thoughtful comments.

However, the reviewers and editor have identified important issues that need to be addressed, some of them related to figures numbering, citations and, particularly, to the writing style. See specific comments below:

1. Figures: There are no numbers on the figures - they should be included and well cited in the text.

Figure numbers were added to the top of each figure.

2. Citations: In a few places in the manuscript, the citations could be more accurate. Currently, some of the citations are not reflecting the results and conclusions of the cited paper. I will point out one, and I would like to invite authors to carefully evaluate the accuracy of each citation. Here is one instance:

Line 92-3: It was then shown that the extent of the dopamine response corresponds to predicted reward magnitude (Bayer and Glimcher, 2005, ...)

Bayer and Glimcher, 2005 explicitly states that they found no such relation. From their paper: “We found no evidence that this signal predicted the magnitude of the upcoming reward, a fact which likely reflects the temporal uncertainties of our particular task (Schultz et al., 1997).”

We carefully edited our citations in the revised manuscript.

3. Writing style: A critical limiting factor of this article is the serious deficits in the writing style. The descriptions are obscure, there are insufficient explanations (as if the authors had not understood their study, although I am sure they did), and the whole text is un-understandable for both the specialist and the non-specialist, both for economics and neuroscience.

The authors can use the comments below regarding the Results sections as guidelines for improving also the rest of the paper (note that specific comments on those other sections are not provided, even though careful revision of those is also needed). Before submitting a revision, the authors should make sure that the new version is understandable and following very good scientific writing standards.

We agree that we failed to sufficiently explain economic theory and omitted important neuroscientific clarifiers as well. Our changes appear in the revision in blue type face.

Abstract:

A small detail: To make a paper on both reward and punishment more understandable, the term ‘prediction error’ without indicating ‘reward’ or ‘punishment’ is unclear: the terms positive or negative prediction error should be replaced by positive or negative reward prediction error, or by positive or negative punishment prediction error, depending on what you mean.

Positive/negative clarifiers were added throughout the manuscript.

After the considerations stated below, I might understand the sentence ‘Increasing release at avoidance made animals less sensitive to price, consistent with a positive prediction error’ (lines 69-70) as saying that optogenetic dopamine stimulation adds positive value to whatever happens at that moment, which may well reduce the negative value of price (and thus make animals ‘less sensitive to price’). Is that correct? But I don't understand what the sentence means “Increasing release at an avoidance predictive cue made animals more sensitive to price, consistent with a negative prediction error” (lines 69-70). Would the negative PE be at the cue, or at the avoidance? In the latter case, optogenetically, artificially increased dopamine would generate enhanced value at the cue, which then could lead to a negative PE at the not-value-enhanced avoidance. Is that meant? Please say so.

The editor is correct; in the revised abstract we added clarifiers in simple generalizable terms in parentheses.

Introduction: The part concerning dopamine and avoidance is made unnecessarily complicated and unclear. The dopamine release with avoidance may well reflect the relief derived from the avoidance, and behavioral studies have shown that this relief has positive reinforcing properties. Thus, a dopamine increase with avoidance, or even the prediction of avoidance, may be a simple positive reinforcement signal. That function would not be properly captured by the general wording of ‘a general role for dopamine in avoidance’ (line 106). Phrased more precisely would make the message of the paper much clearer. Of course, that argument may reflect only one of several possible interpretations, and if there are alternative reasonable interpretations, they should be stated clearly as well.

We removed this confusing statement and revised the paragraph describing previous studies investigating the role of dopamine in avoidance.

The wording on lines 121-123 is entirely un-understandable: what does it mean in terms of reward value if ‘dopamine release at avoidance-related cues should scale with avoidance costs’? Should the dopamine release go up or down with increasing cost (scaled positively or negatively; dopamine should scale negatively with cost=negative reward)? Just say it, and then say clearly what this directional change would mean in terms of dopamine value coding. Maybe you could explain this in terms of ‘worth an animal places on an outcome’ mentioned above. Once we understand this sentence, we can search where the reward prediction error comes in and in which direction it should go. Further, the sentence starting at line 124 is perfectly un-understandable.

We revised and clarified this entire section.

Maybe it would help if the authors could define / explain at the outset whether price and demand have positive or negative reward value, each one separately. Price (= effort, energy expenditure, like lever pressing) should be cost (and dopamine concentration goes down accordingy (fig 4), but what about demand? With increasing price, theoretically demand should drop, so they are inversely related (although this relationship was found only in parts of the behavioral data). Would then ‘increasing the demand to avoid’ be indicative of a price drop = increased value? This is a huge step, with plenty of assumptions, and not taking it apart and describing it well does not help. In fact, I could not find anywhere a definition of how demand was measured: was it the avoidance / unit-price shown in fig 1? It should be the number of avoided shocks, or the intensity of the avoidance in terms of lever presses to be compatible with standard definitions for demand (which one did you measure? Please state at beginning of Results section). Wikipedia says for demand ‘the amount that consumers are willing and able to purchase’ - such a statement should be repeatedly made throughout the paper (and we should not need to go to Wikipedia when reading a paper).

We added descriptions of demand theory in both the results (360) and discussion (622). We regret failing to explain these critically important concepts in our original submission.

What does avoidance / unit-price stand for: is this a ratio as the ‘/’ symbol indicates: avoidance divided by unit-price (?), or does this mean simply avoidance at each unit-price (which would be correct)? The text (line 388) seems to say the latter, but the y-axis label in fig 3a would then be misleading.

We relabeled our axes as "avoidance at each price" throughout the manuscript.

It would be helpful if the authors could describe what fig 3a shows. I think Reviewer 1 got it wrong when stating ‘rats avoid less for lowest avoidance price compared to the second price’, as this would violate the law of price and demand, as shown in fig 3a (the lower the price, the higher the demand). A succinct description could help before going into sidelines about learning effects.

Reviewer 1 is correct. A unique feature of within session behavioral economics sessions is that, after substantial training, demand and dopamine are both concurrently attenuated at session onset (lines: 432-447). This initial attenuation develops over the course of training. The attenuated response and its association with dopamine concentration is independently described in detail in figure 10 and lines 576-598.

## References

[B1] Arnt J (1982) Pharmacological specificity of conditioned avoidance response inhibition in rats: inhibition by neuroleptics and correlation to dopamine receptor blockade. Acta Pharmacol Toxicol (Copenh) 51:321–329. 10.1111/j.1600-0773.1982.tb01032.x 6129770

[B2] Badrinarayan A, Wescott S, Vander Weele C, Saunders B, Couturier B, Maren S, Aragona B (2012) Aversive stimuli differentially modulate real-time dopamine transmission dynamics within the nucleus accumbens core and shell. J Neurosci 32:15779–15790. 10.1523/JNEUROSCI.3557-12.2012 23136417 PMC3752139

[B3] Bentzley BS, Jhou TC, Aston-Jones G (2014) Economic demand predicts addiction-like behavior and therapeutic efficacy of oxytocin in the rat. Proc Natl Acad Sci USA 111:11822–11827. 10.1073/pnas.1406324111 25071176 PMC4136574

[B4] Brischoux F, Chakraborty S, Brierley DI, Ungless MA (2009) Phasic excitation of dopamine neurons in ventral VTA by noxious stimuli. Proc Natl Acad Sci USA 106:4894–4899. 10.1073/pnas.0811507106 19261850 PMC2660746

[B5] Chaudhury D, Walsh JJ, Friedman AK, Juarez B, Ku SM, Koo JW, Ferguson D, Tsai HC, Pomeranz L, Christoffel DJ, Nectow AR, Ekstrand M, Domingos A, Mazei-Robison MS, Mouzon E, Lobo MK, Neve RL, Friedman JM, Russo SJ, Deisseroth K, et al. (2013) Rapid regulation of depression-related behaviours by control of midbrain dopamine neurons. Nature 493:532–536. 10.1038/nature11713 23235832 PMC3554860

[B6] Darvas M, Fadok J, Palmiter R (2011) Requirement of dopamine signaling in the amygdala and striatum for learning and maintenance of a conditioned avoidance response. Learn Mem 18:136–143. 10.1101/lm.2041211 21325435 PMC3056517

[B7] Ebner S, Roitman M, Potter D, Rachlin A, Chartoff E (2010) Depressive-like effects of the kappa opioid receptor agonist salvinorin A are associated with decreased phasic dopamine release in the nucleus accumbens. Psychopharmacology 210:241–252. 10.1007/s00213-010-1836-5 20372879 PMC2894632

[B8] Enomoto K, Matsumoto N, Nakai S, Satoh T, Sato TK, Ueda Y, Inokawa H, Haruno M, Kimura M (2011) Dopamine neurons learn to encode the long-term value of multiple future rewards. Proc Natl Acad Sci USA 108:15462–15467. 10.1073/pnas.1014457108 21896766 PMC3174584

[B9] Fiorillo C (2013) Two dimensions of value: dopamine neurons represent reward but not aversiveness. Science 341:546–549. 10.1126/science.1238699 23908236

[B10] Floresco SB (2015) The nucleus accumbens: an interface between cognition, emotion, and action. Annu Rev Psychol 66:25–52. 10.1146/annurev-psych-010213-115159 25251489

[B11] Fortin S, Chartoff E, Roitman M (2016) The aversive agent lithium chloride suppresses phasic dopamine release through central GLP-1 receptors. Neuropsychopharmacology 41:906–915. 10.1038/npp.2015.220 26211731 PMC4707837

[B12] Fragale JE, Beck KD, Pang KC (2017) Use of the exponential and exponentiated demand equations to assess the behavioral economics of negative reinforcement. Front Neurosci 11:77. 10.3389/fnins.2017.00077 28270744 PMC5318419

[B13] Geist TD, Ettenberg A (1997) Concurrent positive and negative goalbox events produce runway behaviors comparable to those of cocaine-reinforced rats. Pharmacol Biochem Behav 57:145–150. 9164565 10.1016/s0091-3057(96)00300-0

[B14] Gentry RN, Lee B, Roesch MR (2016) Phasic dopamine release in the rat nucleus accumbens predicts approach and avoidance performance. Nat Commun 7:13154. 10.1038/ncomms13154 27786172 PMC5095290

[B15] Glimcher PW (2011) Understanding dopamine and reinforcement learning: the dopamine reward prediction error hypothesis. Proc Natl Acad Sci USA 108:15647–15654. 10.1073/pnas.1014269108 21389268 PMC3176615

[B16] Graybiel A, Grafton S (2015) The striatum: where skills and habits meet. Cold Spring Harb Perspect Biol 7:a021691. 10.1101/cshperspect.a021691 26238359 PMC4526748

[B17] Haber S (2014) The place of dopamine in the cortico-basal ganglia circuit. Neuroscience 282:248–257. 10.1016/j.neuroscience.2014.10.008 25445194 PMC5484174

[B18] Jhou TC, Fields HL, Baxter MG, Saper CB, Holland PC (2009) The rostromedial tegmental nucleus (RMTg), a GABAergic afferent to midbrain dopamine neurons, encodes aversive stimuli and inhibits motor responses. Neuron 61:786–800. 10.1016/j.neuron.2009.02.001 19285474 PMC2841475

[B19] Jin X, Tecuapetla F, Costa R (2014) Basal ganglia subcircuits distinctively encode the parsing and concatenation of action sequences. Nat Neurosci 17:423–430. 10.1038/nn.3632 24464039 PMC3955116

[B20] Lak A, Stauffer WR, Schultz W (2014) Dopamine prediction error responses integrate subjective value from different reward dimensions. Proc Natl Acad Sci USA 111:2343–2348. 10.1073/pnas.1321596111 24453218 PMC3926061

[B21] Lammel S, Lim BK, Ran C, Huang KW, Betley MJ, Tye KM, Deisseroth K, Malenka RC (2012) Input-specific control of reward and aversion in the ventral tegmental area. Nature 491:212–217. 10.1038/nature11527 23064228 PMC3493743

[B22] Lammel S, Lim BK, Malenka RC (2014) Reward and aversion in a heterogeneous midbrain dopamine system. Neuropharmacology 76:351–359. 10.1016/j.neuropharm.2013.03.019 23578393 PMC3778102

[B23] Matsumoto H, Tian J, Uchida N, Watabe-Uchida M (2016) Midbrain dopamine neurons signal aversion in a reward-context-dependent manner. Elife 5:e17328. 10.7554/eLife.17328 27760002 PMC5070948

[B24] Matsumoto M, Hikosaka O (2009) Two types of dopamine neuron distinctly convey positive and negative motivational signals. Nature 459:837–841. 10.1038/nature08028 19448610 PMC2739096

[B25] McCullough LD, Sokolowski JD, Salamone JD (1993) A neurochemical and behavioral investigation of the involvement of nucleus accumbens dopamine in instrumental avoidance. Neuroscience 52:919–925. 8450978 10.1016/0306-4522(93)90538-q

[B26] McCutcheon JE, Ebner SR, Loriaux AL, Roitman MF (2012) Encoding of aversion by dopamine and the nucleus accumbens. Front Neurosci 6:137. 10.3389/fnins.2012.00137 23055953 PMC3457027

[B27] Mileykovskiy B, Morales M (2011) Duration of inhibition of ventral tegmental area dopamine neurons encodes a level of conditioned fear. J Neurosci 31:7471–7476. 10.1523/JNEUROSCI.5731-10.2011 21593330 PMC3153414

[B28] Navratilova E, Porreca F (2014) Reward and motivation in pain and pain relief. Nat Neurosci 17:1304–1312. 10.1038/nn.3811 25254980 PMC4301417

[B29] Ögren SO, Stiedl O (2010) Passive avoidance In: Encyclopedia of psychopharmacology (Stolerman IP, ed), pp 990-996. Berlin, Heidelberg: Springer.

[B30] Oleson EB, Cachope R, Fitoussi A, Tsutsui K, Wu S, Gallegos JA, Cheer JF (2014) Cannabinoid receptor activation shifts temporally engendered patterns of dopamine release. Neuropsychopharmacology 39:1441–1452. 10.1038/npp.2013.340 24345819 PMC3988547

[B31] Oleson EB, Roberts DC (2009) Behavioral economic assessment of price and cocaine consumption following self-administration histories that produce escalation of either final ratios or intake. Neuropsychopharmacology 34:796–804. 10.1038/npp.2008.195 18971927 PMC2626138

[B32] Oleson EB, Richardson JM, Roberts DC (2011) A novel IV cocaine self-administration procedure in rats: differential effects of dopamine, serotonin, and GABA drug pre-treatments on cocaine consumption and maximal price paid. Psychopharmacology 214:567–577. 10.1007/s00213-010-2058-6 21110008 PMC3289955

[B33] Oleson EB, Gentry RN, Chioma VC, Cheer JF (2012) Subsecond dopamine release in the nucleus accumbens predicts conditioned punishment and its successful avoidance. J Neurosci 32:14804–14808. 10.1523/JNEUROSCI.3087-12.2012 23077064 PMC3498047

[B34] Pignatelli M, Bonci A (2015) Role of dopamine neurons in reward and aversion: a synaptic plasticity perspective. Neuron 86:1145–1157. 10.1016/j.neuron.2015.04.015 26050034

[B35] Roitman MF, Wheeler RA, Wightman RM, Carelli RM (2008) Real-time chemical responses in the nucleus accumbens differentiate rewarding and aversive stimuli. Nat Neurosci 11:1376–1377. 10.1038/nn.2219 18978779 PMC3171188

[B36] Schelp SA, Pultorak KJ, Rakowski DR, Gomez DM, Krzystyniak G, Das R, Oleson EB (2017) A transient dopamine signal encodes subjective value and causally influences demand in an economic context. Proc Natl Acad Sci USA 114:E11303–E11312. 10.1073/pnas.1706969114 29109253 PMC5748169

[B37] Schelp SA, Brodnik ZD, Rakowski DR, Pultorak KJ, Sambells AT, España RA, Oleson EB (2018) Diazepam concurrently increases the frequency and decreases the amplitude of transient dopamine release events in the nucleus accumbens. J Pharmacol Exp Ther 364:145–155. 10.1124/jpet.117.241802 29054857 PMC5741045

[B38] Schultz W, Dayan P, Montague PR (1997) A neural substrate of prediction and reward. Science 275:1593–1599. 9054347 10.1126/science.275.5306.1593

[B39] Schultz W, Carelli RM, Wightman RM (2015) Phasic dopamine signals: from subjective reward value to formal economic utility. Curr Opin Behav Sci 5:147–154. 10.1016/j.cobeha.2015.09.006 26719853 PMC4692271

[B40] Schultz W, Stauffer WR, Lak A (2017) The phasic dopamine signal maturing: from reward via behavioural activation to formal economic utility. Curr Opin Neurobiol 43:139–148. 10.1016/j.conb.2017.03.013 28390863

[B41] Sidman M (1953) Avoidance conditioning with brief shock and no exteroceptive warning signal. Science 118:157–158. 13076224 10.1126/science.118.3058.157

[B42] Stauffer WR, Lak A, Schultz W (2014) Dopamine reward prediction error responses reflect marginal utility. Curr Biol 24:2491–2500. 10.1016/j.cub.2014.08.064 25283778 PMC4228052

[B43] Steinberg EE, Keiflin R, Boivin JR, Witten IB, Deisseroth K, Janak PH (2013) A causal link between prediction errors, dopamine neurons and learning. Nat Neurosci 16:966–973. 10.1038/nn.3413 23708143 PMC3705924

[B44] Tan KR, Yvon C, Turiault M, Mirzabekov JJ, Doehner J, Labouèbe G, Deisseroth K, Tye KM, Lüscher C (2012) GABA neurons of the VTA drive conditioned place aversion. Neuron 73:1173–1183. 10.1016/j.neuron.2012.02.015 22445344 PMC6690362

[B45] Tobler PN, Fiorillo CD, Schultz W (2005) Adaptive coding of reward value by dopamine neurons. Science 307:1642–1645. 10.1126/science.1105370 15761155

[B46] Tye KM, Mirzabekov JJ, Warden MR, Ferenczi EA, Tsai H, Finkelstein J, Kim S, Adhikari A, Thompson RK, Andalman AS, Gunaydin LA, Witten IB, Deisseroth K (2013) Dopamine neurons modulate neural encoding and expression of depression-related behaviour. Nature 493:537–541. 10.1038/nature11740 23235822 PMC4160519

[B47] Ungless MA, Grace AA (2012) Are you or aren’t you? Challenges associated with physiologically identifying dopamine neurons. Trends Neurosci 35:422–430. 10.1016/j.tins.2012.02.003 22459161 PMC3383926

[B48] Van Rijn H, Gu BM, Meck WH (2014) Dedicated clock/timing circuit theories of time perception and timed performance. Adv Exp Med Biol 829:75–99. 10.1007/978-1-4939-1782-2_5 25358706

[B49] Wadenberg M, Ericson E, Magnusson O, Ahlenius S (1990) Suppression of conditioned avoidance behavior by the local application of (−)sulpiride into the ventral, but not the dorsal, striatum of the rat. Biol Psychiatry 28:297–307. 10.1016/0006-3223(90)90657-N 2144458

[B50] Willuhn I, Tose A, Wanat MJ, Hart AS, Hollon NG, Phillips PE, Schwarting RK, Wöhr M (2014) Phasic dopamine release in the nucleus accumbens in response to pro-social 50 kHz ultrasonic vocalizations in rats. J Neurosci 34:10616–10623. 10.1523/JNEUROSCI.1060-14.2014 25100595 PMC4200110

